# Prolonged QT interval and cardiac arrest after a single dose of amiodarone in a woman with Turner's syndrome

**DOI:** 10.1002/ccr3.802

**Published:** 2017-01-21

**Authors:** Dorte Guldbrand Nielsen, Jens Cosedis Nielsen, Christian Trolle, Claus Højbjerg Gravholt, Niels Holmark Andersen

**Affiliations:** ^1^ Department of CardiologyAarhus University HospitalAarhus NDK‐8200Denmark; ^2^ Department of Endocrinology and Internal MedicineAarhus University HospitalAarhus CDK‐8000Denmark

**Keywords:** Amiodarone, arrhythmia, cardiac arrest, QTc prolongation, Turner's syndrome

## Abstract

Low‐dose QT‐prolonging drugs may have detrimental effects on women with Turner's syndrome. Preventive measures would be to use potential QT‐prolonging drugs with precaution and ensure that both before and during treatment, ECGs are evaluated and drug treatment stopped if the QT interval increases.

## Introduction

The standardized mortality risk for the adult patient with Turner's syndrome is significantly higher than for the general population 1. Causes of cardiovascular death in the adult Turner population include aortic dissection and coronary artery disease. However, a significant number of patients with Turner's syndrome die suddenly due to unexplained causes 1, 2. A number of these unexplained deaths have been explained as unspecified seizures, but might have been caused by undiagnosed cardiac arrhythmia.

Treatment with QT prolongation is well documented in patients with Turner's syndrome, but the clinical significance is still uncertain 3, 4. New data indicate that there is a connection between human estrogen levels and QT duration, where high levels of estradiol were associated with shorter QTc intervals in healthy women due to effects on KCNH2 receptors in the cell membrane 5. Conversely, this may explain why some patients with Turner's syndrome and low estrogen levels may have prolonged QT intervals 3, 4. It has also been found that a rather high number of patients with Turner's syndrome carry variants in the long QT genes, including the SCN5A and KCNH2 genes 6. This will obviously add to the prevalence of QT prolongation in a Turner cohort.

QT prolongation in patients with Turner's syndrome may have clinical consequences, so special precautions may be necessary when using QT‐prolonging drugs, also despite the lack of specific recommendations in this area. A recent single case study described a 20‐year‐old female with Turner's syndrome, who died suddenly. The autopsy revealed a malignant ovarian teratoma, but without metastases, the cause of death was described as uncertain. However, the woman had been treated with the potentially QT‐prolonging drug 7, quetiapine, in therapeutic doses at the time of death 7. This drug in combination with QT prolongation associated with Turner's syndrome may have induced ventricular tachyarrhythmia and subsequent death.

At present, there is not much literature available about this subject and therefore not enough evidence to issue a general recommendation. This makes it even more important to report all cases where patients with Turner's syndrome react abnormally when treated with potentially QT‐prolonging drugs.

In this present case, one single dose of 300 mg amiodarone turned out to have life‐threatening effects for an adult woman with Turner's syndrome.

## Case

**Figure 1:**
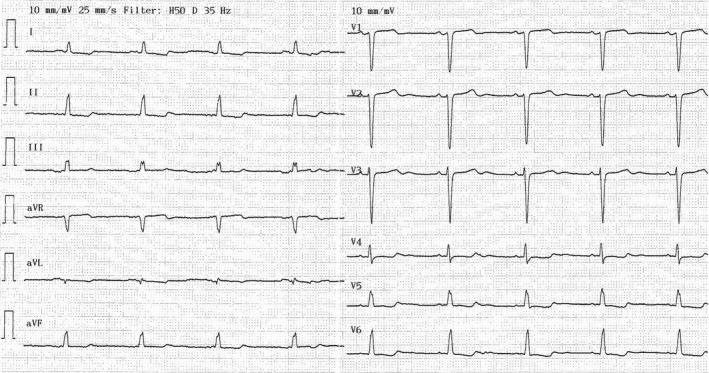
ECG obtained the year before the current admission. The QTc as estimated by Fridericia correction formula is 465 msec (heart rate 55 beats per min and QT 480 msec).

A 58‐year‐old woman with Turner's syndrome (karyotype 45,X0), type 2 diabetes, and heart failure was referred to our hospital because of shortness of breath. Her heart rhythm was irregular, and a diagnosis of atrial fibrillation was confirmed by pathognomonic electrocardiographic findings. The year before, to rule out a suspected coronary heart disease, she had been carefully examined by means of ECG (Fig. 1), echocardiogram, 24‐h ECG monitoring, and coronary angiogram. At that time, her ejection fraction was measured to approx. 35%, her coronary arteries were normal, and QTc was 465 msec (Fridericia correction formula). She was normal weight and not treated with estrogen therapy. After approximately 10 months of treatment with metoprolol 50 mg b.i.d, enalapril 20 mg o.d., and spironolactone 25 mg o.d., her ejection fraction had improved to around 50%. Furthermore, because of her previous, short episodes of atrial fibrillation, treatment with dabigatran 110 mg b.i.d. was initiated.

**Figure 2:**
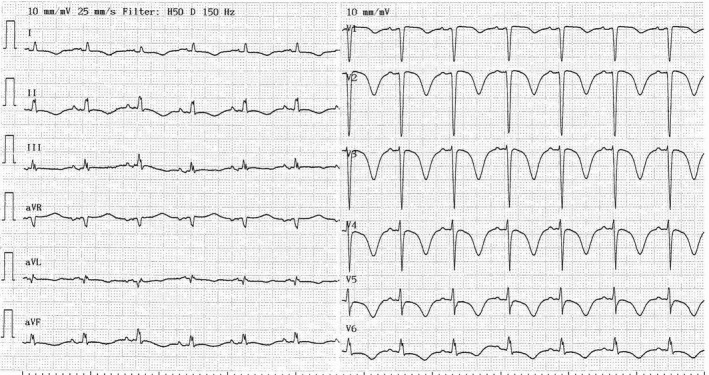
ECG obtained after cardioversion. The QTc as estimated by Fridericia correction formula is 608 msec (heart rate 77 beats per min and QT interval 560 msec).

**Figure 3:**
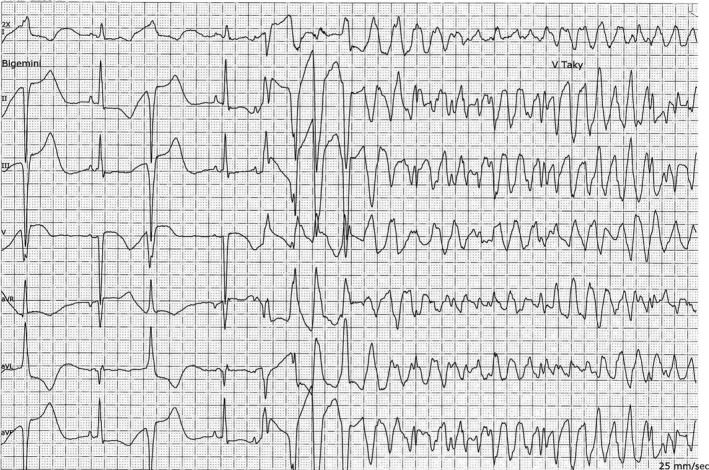
ECG with severely prolonged QT interval and initiation of fast polymorphic ventricular tachycardia degenerating into ventricular fibrillation.

Upon admission, the patient showed shortness of breath and irregular heart rhythm. A new ECG revealed that she had atrial fibrillation. It was decided to perform a subacute cardioversion, and prior to this, she was given a single dose of intravenous amiodarone (300 mg). Her electrolytes were normal, and she was not treated with any other QT‐prolonging drugs. After 12 h, she was successfully cardioverted. After the procedure, however, the ECG showed considerable changes, including significant QT prolongation (600 msec: Fig. 2) and negative T‐waves. As a consequence, she was kept under continuous ECG monitoring. Approximately 17 h after the amiodarone infusion, she developed cardiac arrest with ventricular fibrillation (Fig. 3). She was successfully resuscitated by immediate cardioversion and received no additional amiodarone treatment. The ECG normalized after <2 weeks.

Afterward, the patient was tested for variants in the long QT genes, including KCNE1, KCNE1L, KCNE2, KCNH2, KCNJ2, KCNQ1, RYR2, SCN4B, SCNSA, and SNTA1. All tests were negative.

## Discussion

This case shows that a woman with Turner's syndrome and with only mildly prolonged QT interval could develop life‐threatening arrhythmia following even a very low dose of amiodarone. The patient was not taking any other QT‐prolonging drugs, and therefore, treatment with amiodarone at such a low dose and with such short exposure time 8 should not have been able to cause QT prolongation to a life‐threatening level.

The QT interval seems to have a relation to the number of sex chromosomes in a given individual. With regard to patients with Turner's syndrome, where the majority of women have the 45,X karyotype, that is, they lack a X chromosome, the tendency is toward QT prolongation 6, whereas with regard to the Klinefelter's syndrome (a condition describing males with an extra X chromosome, 47,XXY), the QT interval has been found to be shorter than for males without this condition 9. The common pathway could be that it is due to, either the number of sex chromosomes or more likely an interplay between testosterone and estrogen, or possibly a combination of genes on the X chromosomes and the sex hormones 10. Since both estradiol and testosterone shorten the QT interval, the implication for both Turner's and Klinefelter's syndrome may be hormonal. Endogenous testosterone or testosterone treatment may be the primary reason for short QT intervals in Klinefelter patients, whereas in patients with Turner's syndrome, the mechanisms may be more complex and multifactorial. Prolongation of the QT interval may be influenced by factors, such as X‐chromosomal haploinsufficiency, genetics, estrogen deficiency, and dosage of hormone replacement or increased sympathetic activation 11. Because as many as 33% of Turner women (45X0) have a prolonged QT interval 6, it seems likely that genetics may somehow be involved. However, till now, scientists have not been able to establish any gene or genetic connection.

The woman in the present case did no longer receive estrogen replacement therapy. Estrogen treatment may have a beneficial effect on a prolonged QT interval. The dose of estradiol given to females with Turner's syndrome may vary with the patient's age, where the dose is slowly escalated during puberty, typically approaching 2 mg of 17 *β*‐estradiol. During adulthood, the typical dose is 2‐3‐4 mg orally. However, there is no consensus regarding the optimal dose during the different stages of life. As the level of estradiol modulates the QT interval, the 17*β*‐estradiol dose should be influenced by the ambient QT interval in a given patient. QT‐prolonging drugs indiscriminately may be fatal in patients with Turner's syndrome, which this present case also clearly demonstrates.

## Conclusion

Even a low dose of QT‐prolonging drugs may have detrimental effects on a patient with Turner's syndrome. Preventive measures should be taken to use potential QT‐prolonging drugs with precaution and ensure that ECGs are evaluated both before and during treatment, and drug treatment stopped, if the QT interval increases. By careful drug selection and close monitoring, if a specific drug is required, the risk of sudden cardiac death in patients with Turner's syndrome may be significantly reduced.

## Conflict of Interest

None of the authors have any conflict of interest to declare.

## Authorship

DGN: collected data and wrote first draft of article together with NHA, was in charge of the final writing process. JCN: analyzed all ECGs and calculated QTc prolongation, participated in the final writing process. CT: contributed with expert knowledge of Turner's syndrome and participated in the writing of the article. CHG: contributed with expert knowledge of Turner's syndrome and participated in the writing of the article. NHA: collected data and wrote the first draft of the article together with DGN, participated in the final writing process.
